# Nanoscale Printing of Indium-Tin-Oxide by Femtosecond Laser Pulses

**DOI:** 10.3390/nano12224092

**Published:** 2022-11-21

**Authors:** Jingwen Hu, Zhen-Ze Li, Yang-Yang Zhao, Yi-Shi Xu, Lin Wang, Molong Han, Lachlan Hyde, Soon Hock Ng, Lei Wang, Saulius Juodkazis

**Affiliations:** 1 Optical Sciences Centre and ARC Training Centre in Surface Engineering for Advanced Materials (SEAM), School of Science, Swinburne University of Technology, Hawthorn, VIC 3122, Australia; 2 State Key Laboratory of Integrated Optoelectronics, College of Electronic Science and Engineering, Jilin University, Changchun 130012, China; 3 Melbourne Centre for Nanofabrication (MCN-ANFF), 151 Wellington Rd, Clayton, VIC 3168, Australia; 4 Space Technology and Industry Institute, Graphene Certification Labs, Swinburne University of Technology, Hawthorn, VIC 3122, Australia; 5 World Research Hub Initiative (WRHI), School of Materials and Chemical Technology, Tokyo Institute of Technology, 2-12-1, Ookayama, Meguro-ku, Tokyo 152-8550, Japan

**Keywords:** IR, laser printing, ITO, solid resist

## Abstract

For constructing optical and electrical micro-devices, the deposition/printing of materials with sub-1 μm precision and size (cross-section) is required. Crystalline c-ITO (indium tin oxide) nanostructures were patterned on glass with sufficient precision to form 20–50 nm gaps between individual disks or lines of ∼250 nm diameter or width. The absorbed energy density [J/cm${}^{3}$] followed a second-order dependence on pulse energy. This facilitated high-resolution and precise nanoscale laser-writing at a laser wavelength of 515 nm. Patterns for optical elements such as circular gratings and micro-disks were laser-printed using ITO as a resist. Unexposed amorphous a-ITO was chemically removed in aqueous 1% vol. HF solution. This use of a-ITO as a solid resist holds promise for metamaterial and micro-optical applications.

## 1. Introduction

Indium tin oxide (ITO) is an n-type semiconductor with a 3.5–3.7 eV bandgap. ITO is transparent under visible light in the 390 nm to 700 nm wavelength range (3.2 to 1.8 eV) [1,2], has a refractive index of n≈1.5, and an extinction coefficient of κ<0.005 [3]. In addition to its excellent optical properties, ITO also has useful and unique electrical properties, namely, low electrical resistivity $\rho \approx 10^{-5}\;\Omega .$cm. Regarding indium oxide, oxygen vacancies contribute large quantities of native donors. When highly doped with tin (more than 10 at.%), oxygen vacancies no longer function as predominating defects. Sn will donate an electron, which leads to the dominant conduction mechanism: ${\mathrm{Sn}}^{4+}$ substitutes the position of ${\mathrm{In}}^{3+}$ resulting in the addition of free electrons [4,5]. A high-carrier concentration from the Sn donor and large mobility lead to low film resistivity, approximating $<10^{-4}$ Ω cm [6]. Because of its low-cost fabrication potential, ITO is currently widely used in transparent display devices, transparent coatings for photovoltaics such as solar cells, organic light-emitting devices (OLEDs), photodiodes, and phototransistors due to its high transmittance and relatively high conductivity [7,8,9]; with an electron density of $n_{e}$∼$10^{19}$ cm${}^{-3}$. In the near-IR spectral window of λ = 1.1–1.3 μm, ITO is an epsilon-near-zero (ENZ) $\varepsilon \equiv {\left(n+i\kappa \right)}^{2}\to 0$ material [10]. The ENZ condition opens up possibilities of tailoring energy deposition in perfect absorbers and associated exploitation for metamaterial applications and nonlinear optics [11,12,13,14,15,16]. The highest transmission of 2D ITO T>99% and sheet resistance of $R_{S}\equiv \rho /t=5.4$ kΩ/□ [17] was demonstrated using low-temperature liquid-metal synthesis [18]; *t* is the thickness of a resistor film. This opens up greater opportunities for flexible electronics and optics, including wearables and sensors [19,20].

It is established that the electrical properties of ITO films rely on the film deposition and annealing conditions. The amorphous (a-ITO) and crystalline (c-ITO) phases of ITO bear different resistivities, owing to the lattice arrangement varying the carrier density [21]. It was shown that ITO can be used as a negative tone resist by writing patterns in glass using a sub-15 fs laser and subsequent etching in 10% hydrochloric acid solution [22]. Sub-1 μm-wide ITO lines down to 50 nm were demonstrated by scanning a focused fs-laser beam [22,23,24,25]. Large-sized ripple structures were also fabricated by controlling the polarisation in concert with the writing direction [26,27,28]. In these experiments, the obtained patterns were either of poor quality or unable to be manufactured flexibly and precisely. Previous studies have shown that laser irradiation of a-ITO induces crystallisation [23,29,30,31]. These c-ITO regions exhibit varying selectivity to the a-ITO, etching more slowly in both HCl and Fe_3_-HCl etchants [29,32,33]. Recently, the use of nano-films as a solid resist in combination with laser direct-writing for negative and positive tones has attracted interest [34,35].

In this study, amorphous a-ITO films were modified via sub-1 ps laser pulses with deep-sub-wavelength precision and resolution. Nanoscale modification via localised annealing and structural modification/crystallisation leading to c-ITO was demonstrated. By meticulously controlling the fs-laser fluence below the ablation threshold, we created a modified ITO on a sub-wavelength scale, which could withstand wet etching in an aqueous HF solution. This provides the possibility of directly writing not just lines, but sub-wavelength patterns and individual nano-particles with controlled size and spacing. Nano-disks and arrays with a diameter of ∼250 nm and nanogaps as small as 25 nm were fabricated with high-resolution and controllability, with a focus on metamaterials and micro-optics.

## 2. Results and Discussion

Direct laser-writing/printing of nano-disks/lines were conducted at the second harmonic wavelength of λ=515 nm (photon energy hν=2.407 eV), which lacked an ns-long pedestal pulse background due to the emission of an excitation-diode pulse radiation. This is important since very small pulse energies $E_{p}$∼0.7 nJ were used and long background emission at the fundamental wavelength would cause significant absorption in ITO (In_2_O_3_-SnO_2_). At the laser excitation wavelength, ITO has index $\tilde{n}\equiv 1.8983+i3.6549\times 10^{-3}$ or permittivity $\varepsilon \equiv {\tilde{n}}^{2}=3.6036+i0.013876$ corresponding to the absorption coefficient α=4πκ/λ=891.83 cm${}^{-1}$ [36]. Even with such strong absorption, an estimate of transmittance $T=10^{-\alpha d}\approx 98.8\%$ for a d=60 nm thick ITO film (reflectance is not taken into account, R=0 used for this qualitative estimate). This shows that linear absorbance was negligible and nonlinear light–matter interactions were important for the following laser-writing/printing.

### 2.1. Single Nano-Disks and Pairs

**Figure 1:**
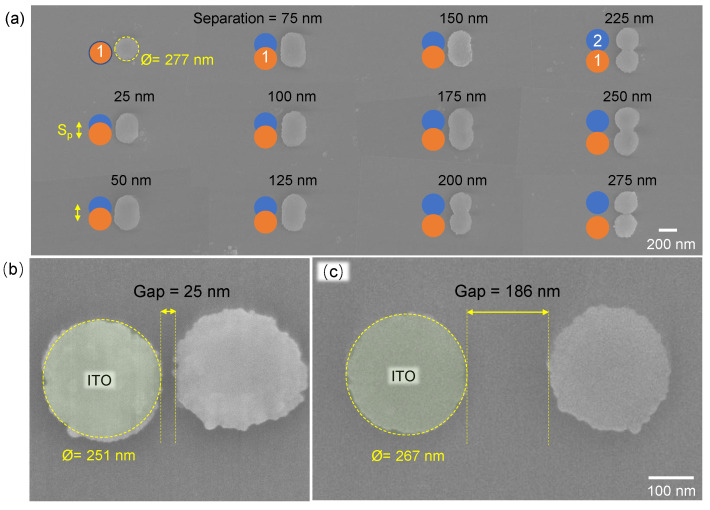
SEM images of ITO structures by nano-printing. (**a**) Two pulse-burst exposure of ITO film after wet etching in 1% vol. HF solution for 5 s. The ITO was printed using two laser-pulse bursts (515 nm/280 fs/10 pulses per burst/ pulse energy $E_{p}=0.92\pm 0.02$ nJ (on sample)) at separations $S_{p}$ varying from 0 to 300 nm. Focusing was performed using an NA=0.9 objective lens; polarisation linear (horizontal). Nanogaps can be controlled from 25 nm (**b**) to a size ten-times larger (**c**). Numerical aperture of the objective lens was NA=0.9 (focal spot diameter ⊘=1.22λ/NA=698 nm), thickness of ITO films d≈60 nm. Polarisation of laser pulses (**b**,**c**) was linear (vertical).

Figure 1a shows scanning electron microscopy (SEM) images of patterned ITO disks made via two laser-pulse bursts of N=10 pulses (230 fs/515 nm) at a laser-pulse energy of $E_{p}=0.92$ nJ and laser-repetition rate of f=1 kHz with subsequent chemical etching. Focus was placed on the 60-nm-thick amorphous a-ITO surface and HF-etching was used to remove unexposed ITO. Two burst positions with separation $S_{p}$ from 0 to 300 nm were used. The irradiated region was recognisable under SEM imaging due to different charging characteristics. However, the disk structure was only revealed after development in aqueous 1% vol. HF solution. Laser-irradiation changed a-ITO and rendered it less soluble in the HF solution. It was shown previously [21] that the deposited laser energy heated the film, annealing a-ITO into polycrystalline c-ITO. This resulted in different etching rates between amorphous and crystalline ITO (Figure 1). A small pulse energy, $E_{p}\approx 0.7$ nJ (and N=10 pulse dose), is required to turn a-ITO in to HF insoluble c-ITO. This energy, focused onto a ⊘≡2r=1.22λ/NA≈700 nm spot, corresponds to a fluence of $F_{p}=E_{p}/\left(\pi r^{2}\right)\approx 0.18$ J/cm${}^{2}$ per pulse and an irradiance (average) of $I_{p}=F_{p}/t_{p}\approx 0.79$ TW/cm${}^{2}$ (where pulse duration was $t_{p}=230$ fs and numerical aperture of the objective lens was NA=0.9). Doubling of the fluence ($E_{p}\approx 1.4$ nJ) was sufficient to ablate 200 nm sub-diffraction holes in the 60-nm-thick ITO film (not shown here).

**Figure 2:**
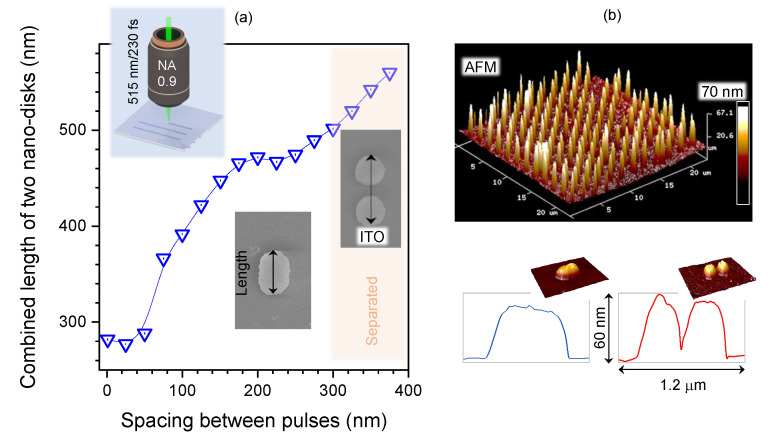
(**a**) Plot showing the length of two pulse-burst-exposed ITO nano-disks vs. separation between these two bursts *S*${}_{p}$ measured via SEM imaging. (**b**) AFM profilometry of laser-printed nano-disks. Two pulse-burst exposure of ITO film after wet etching in HF solution. Polarisation of laser E-field was horizontal, NA=0.9, pulse energy $E_{p}=0.92\pm 0.02$ nJ (on sample), N=10 pulse bursts per disk at f=5 kHz (for the two-spot exposures).

The blue and orange circles in Figure 1a depict the arrangement of two irradiation sites with different separation. The top-left nano-disk 277 nm in diameter was irradiated by a single burst of N=10 pulses. With an increase in spacing $S_{p}$, the outside contour of the nano-disk pattern stretches to an ellipse then to a gourd shape and eventually the two nano-disks become separated completely. A narrow gap 25 nm in width formed between the two nano-disks (Figure 1b). The distance between two nano-disks 250±20 nm in diameter can be well-controlled and the structural length can be defined (Figure 2a). There was no clear elongation of the nano-disks along the orientation of polarisation. This can be attributed to the low anisotropy of heat transfer (by electrons) during the pulse, which can be considerable in homogeneous and crystalline materials [37] or due to the vectorial nature of focusing at high NA [38]. The surface morphology of the dots’ matrix pattern can be defined (Figure 1b). The height of the ITO disks measured approximately 60 nm, which is also the thickness of the a-ITO film. Separation of the nano-disks is evident from AFM cross-sections (Figure 2b).

### 2.2. From Lines with Nano-Gaps to Optical Micro-Elements

**Figure 3:**
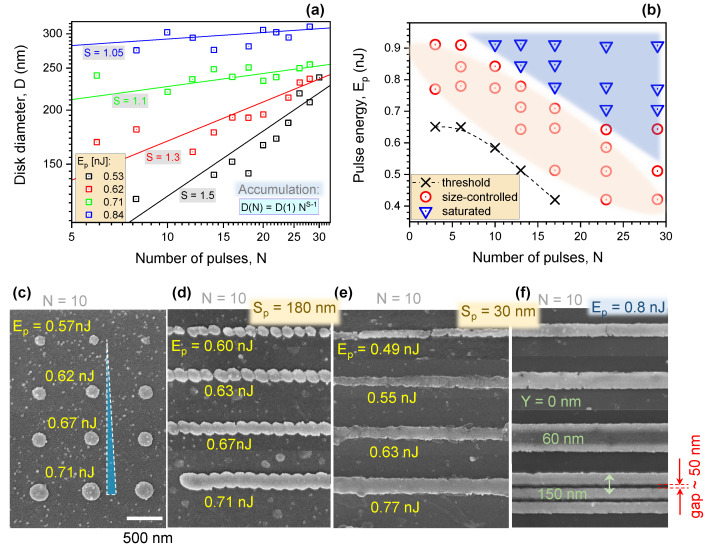
(**a**) Plot showing nano-disk diameter as a function of pulse number *N* measured by SEM for pulse energies between $E_{p}=0.77$ nJ and 0.46 nJ. (**b**) $E_{p}$ vs. *N* diagram for nano-disk fabrication: threshold of disk formation (×), region where different-sized disks can be made (○) and where size (diameter) saturates (▽). (**c**,**d**) c-ITO transitioning from single dots to a line on glass, recorded at different pulse energies $E_{p}$ for N=10 per irradiation site. (**e**) Formation of lines of different widths by selection of $E_{p},N$, and separation $S_{p}$ along the line. (**f**) Pattern of separate lines using vertical *Y* shift. Polarisation linear (horizontal; along the line).

Figure 3a summarises the effect of laser pulse energy $E_{p}$ and dose (via pulse number *N*) for c-ITO pattern formation. By incrementing the number of pulses *N*, the disk diameter increases for $E_{p}=0.55$ nJ until 0.62 nJ. When the pulse energy reaches between 0.71 nJ and 0.84 nJ, the diameter saturates at the maximum size measuring approximately between 240 nm and 300 nm following the fifth pulse, and becomes constant at larger *N*. The pulse energy and pulse number (dose) determine the final diameter *D* (or ⊘) of the nano-disks.

An accumulation effect was present during nano-disk formation. This is where the energy deposited from each successive pulse accumulates, in this case, manifesting as a larger diameter as the number of pulses *N* increases. As usual for accumulation effects, one can apply the dependence $⊘\left(N\right)=⊘\left(1\right)\times N^{S-1}$, where ⊘(1) is the initial disk diameter after N=1 pulses and 0<S≤1 is the accumulation exponent; when S=1 the effect of a multi-pulse exposure has no cumulative effect. The accumulation exponent changed from S=1.5 to 1.05 from the lowest to the largest pulse energies (0.5–0.9 nJ) as shown in Figure 3a. The maximum disk diameter was ⊘= 300 nm, after which it became independent of $E_{p}$ and *N*. A further increase to $E_{p}\geq 0.91$ nJ caused the ablation of a nano-hole. As shown in Figure 3b, three different conditions for nano-disk formation can be distinguished. At the threshold of the $E_{p}$ vs. *N* plot, nano-disks form with the minimum diameter ⊘. The working region, where the diameter can be controlled via the exposure dose $⊘(E_{p},N)$, demonstrates the most utility (circle markers ○).

When $E_{p}=0.46$ nJ, a nano-disk ⊘=100 nm in diameter appears after N>15 pulses and the accumulation effect is strongest (S>1.5).

Figure 3c–f demonstrate flexibility of position and size control over the disk pattern as well as the formation of fine lines of different widths and separation. No c-ITO formed when the pulse energy was lower than 0.46 nJ, even for an N>100 dose (at f=10 kHz repetition rate). The minimum c-ITO nano-disk diameter was approximately 100 nm and the maximum 300 nm. Ablation ripples appeared at the centre of focus for a large dose which presented an undesired result for the dot/line printing. When the distance between adjacent focal spots changed from 30-to-150 nm (along the line), a line pattern developed with wavy-to-smooth edges that was exposure–dose-dependent.

At each exposure site (disk), the laser repetition rate was f=5 kHz and the number of pulses in a burst was N=10. The shutter was closed during travel between each site. Due to the long break between two bursts (scan velocity of $v_{s}=1.15\;\mu$m/s), the ITO modification at a preceding site finished before the next, resulting in a wavy-edged line, dependent on the separation between exposure sites. Polarisation was linear and orientated parallel to the scanning path. The narrowest lines were ∼90 nm close to the smallest diameter of the disks at ∼100 nm. Gaps of ∼50 nm between lines were formed by the described N=10 pulse exposure per irradiation site (Figure 3f).

**Figure 4:**
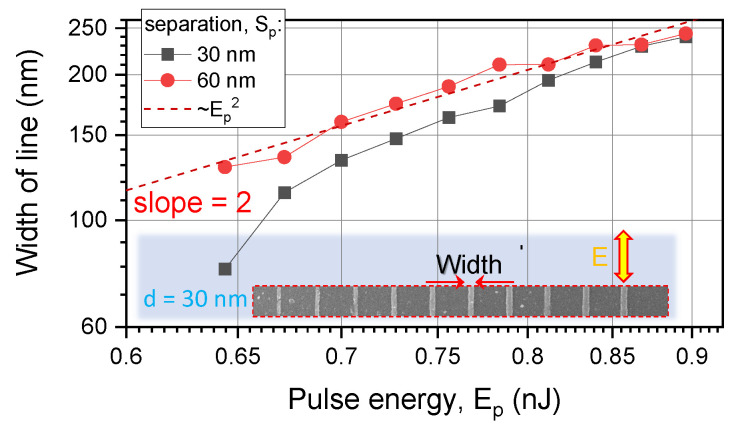
Plot showing the evolution of line-width vs. pulse energy $E_{p}$ for N=10 pulses per site at different separations $S_{p}$ along the line (a log–log plot); polarisation of E-field was linear (along the line) shown in the inset. The trend of dependence closely follows a nonlinear $\propto E_{p}^{2}$ dependence.

Interestingly, the disk diameter was dependent on pulse energy $E_{p}$ in a nonlinear manner, as apparent from Figure 3c. For a line, patterned using the same N=10 pulses per site and with $S_{p}=30,60$ nm, the width closely followed a second power-law-dependence $Width\propto E_{p}^{2}$, as shown in Figure 4. Since pulse energy $E_{p}$, fluence $F_{p}$, dose $D_{p}$, and intensity (irradiance) $I_{p}$ are all proportional to each other, such scaling would imply that nano-disks and lines were formed by two photon absorption (TPA). Indeed, 2hν=4.814 eV is larger than the ITO bandgap of ∼3.7 eV. However, since the initial a-ITO is highly transparent to the 515 nm wavelength used, it was necessary to accumulate several pulses over the same site on 60-nm-thick ITO to render it absorbent. This hints at the importance of absorbed energy density $W_{ab}$ in the volume, i.e., J/cm${}^{3}$ rather than fluence (or dose) J/cm${}^{2}$. When the modification is proportional to instantaneous electron density, $n_{e}$, the deposited-laser-energy density in the volume scales as $W_{ab}\propto \frac{n_{e}}{n_{cr}}F_{p}\propto F_{p}^{2}$, where $n_{cr}$ is the critical electron density at the wavelength of irradiation [39]. This scaling of energy deposition into volume follows a second-order dependence $W_{ab}\propto E_{p}^{2}$, since electron density is proportional to the energy (fluence, intensity) $n_{e}\propto E_{p}$. The strongest energy deposition into the target/sample takes place where a strongly excited region of material (plasma) exists with the electron density approaching the critical; the intensity used in experiment was high ∼1 TW/cm${}^{2}$ and strong ionisation of the material occurs. These are ENZ conditions where material turn into 1>ε>0 (ε=0 is the dielectric breakdown by definition). These conditions of material excitation are also defined as a dielectric-metal (Die-Met) state of matter [13,40].

**Figure 5:**
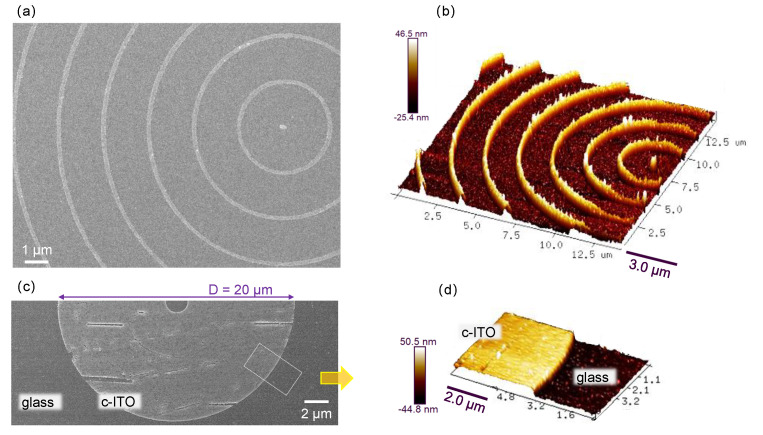
(**a**) SEM image of a Bessel-beam generating a micro-optical element recorded on glass. Polarisation of laser E-field was horizontal, NA=0.9, scanning speed $v_{s}=30\;\mu$m/s and pulse energy $E_{p}=0.9$ nJ (on sample). (**b**) AFM view of the concentric grating with ITO lines that were 60 nm in height. (**c**) Bulk c-ITO recorded on glass. (**d**) AFM view of the bulk of ITO.

Figure 5 demonstrates the micro-optical element fabrication: a Bessel-beam generating concentric grating and a micro-disk optical retarder recorded in c-ITO on glass. The width of a single-line scan measured about 200 nm. A 60 nm height for n≈1.5 corresponds to 23% wave retardance at 400 nm wavelength (close to λ/4). Since the width of the c-ITO line was independent of the linear polarisation orientation, symmetric patterns of the same line-width were formed. An area of a c-ITO disk measuring 20 μm in diameter was formed with small 100 nm radial steps between concentric line scans (Figure 5c,d). Since a large area was exposed during disk fabrication, it was more susceptible to ablation and ripple formation when compared to parallel lines.

### 2.3. Material Analysis

**Figure 6:**
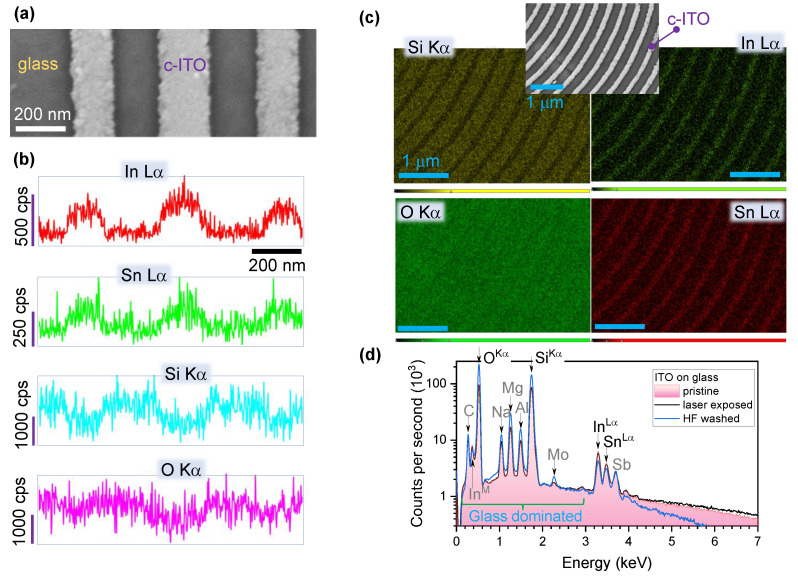
X-ray energy dispersion spectroscopy (EDS) analysis of c-ITO structure. (**a**) SEM image of c-ITO line structure after laser exposure and development. (**b**) Distribution of Si ($K_{\alpha }=1.739$ keV), O ($K_{\alpha }=0.525$ keV), In, Sn according their characteristic lines; 200 nm scale bar is same for all elemental maps. (**c**) Compositional maps of the four elements on the surface of sample. Inset shows SEM image of the mapped surface; all same 1 μm scale bar. (**d**) The EDS spectra of pristine, laser irradiated and HF-developed ITO structures.

Figure 6 summarises characterisation of the C-ITO pattern via energy-dispersive X-ray spectroscopy (EDS). Lines of c-ITO with ∼200 nm gaps obtained after laser printing and HF etching are shown in the SEM image (a). The EDS spectrum (b) shows the corresponding spatial distributions of In, Sn, Si and O measured cross-sectionally and perpendicular to the c-ITO lines. The peaks coincide perfectly with the topography of the ITO lines. In and Sn contribute strongly at the ridge and barely exist between the lines (where the glass substrate is exposed). On the contrary, Si had a significant contribution between c-ITO lines but was nearly negligible on the c-ITO pattern. Oxygen demonstrated a slight increase on the c-ITO lines due to higher concentration in ITO and a cumulative contribution from the greater depths of the SiO_2_-rich glass substrate. Considering that the concentrations of Si and O were an order of magnitude larger than that of In and Sn, a spectral window of EDS spectrum was selected for specific elements. A total of three peaks were expected for the major elements In ($L_{\alpha }=3.286$ keV), Sn ($L_{\alpha }=3.443$ keV) and Sb ($L_{\alpha }=3.604$ keV) which were measured before and after laser exposure as well as after HF etching. Without HF etching, no significant difference was found between pristine and laser-exposed samples. Chih W. et al. reported that the breaking of In-O bonds leads to an increase in In-In bonds and causes In metal-like clusters [41]. Interestingly, the Sn/In ratio of the modified c-ITO pattern area (after HF development) increased by 50% as compared to the non-treated sample. The increase in Sn/In ratio, and hence the loss of In was observed with Fe_3_-HCl etchant and magnetron sputtered then annealed ITO. This was attributed to preferential etching of In_2_O_3_ due to the negative Gibbs free energy of the In${}^{3+}$ dissociation [33]. The lower bond strength of In-O (≈3.73 eV) compared to Sn-O (≈5.68 eV) may be broken by multiphoton absorption [42]. These effects likely affect the etch rates during HF exposure; however, a dedicated evaluation is outside the scope of this discussion.The low-energy side of EDS (Figure 6d) shows larger intensity of peaks after a-ITO is removed, which is an expected result since more glass substrate was exposed and cleaned due to the HF etching.

### 2.4. Numerical Modeling of Light Field Enhancement

**Figure 7:**
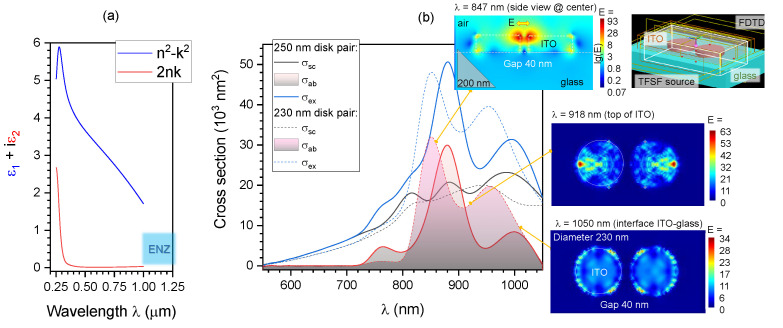
Numerical modeling of light intensity on an ITO metasurface consisting of nano-disk pairs. (**a**) Permittivity $\tilde{\varepsilon }\equiv \varepsilon_{1}+i\varepsilon_{2}\equiv {\left(n+ik\right)}^{2}$ of ITO [36]. The epsilon-near-zero (ENZ) region $0<\varepsilon_{1}<1$ is the near-IR spectral region. (**b**) Cross-sections of scattering, absorption and extinction $\sigma_{ex}\equiv \sigma_{ab}+\sigma_{sc}$ for a pair of ITO nanodisks calculated for 250 nm and 230 nm diameters using a total-field scattered-field (TFSF) source in Lumerical. Refractive index of glass n=1.4, the height of disk h=80 nm, gap 40 nm. Insets show characteristic light enhancement maps at different cross-sections and wavelengths. Linear polarisation of light was aligned perpendicular to the gap.

**Figure A1:**
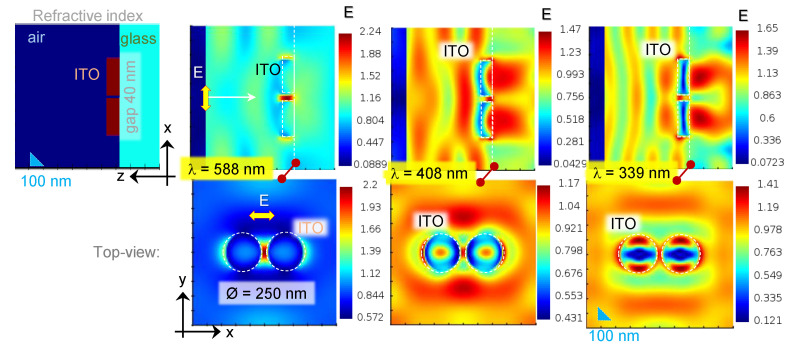
FDTD calculation of light-intensity distribution (within 1μm${}^{3}$ volume) for an array of ITO nano-disk pairs; period in XY-plane is $\Lambda_{x,y}=1\;\mu$m, diameter of ITO disk 2r=250 nm, the gap Δx=40 nm, height of disk h=80 nm. Light source is a plane wave with a normalised E-field ${\left|E\right|}^{2}=1$. Glass is defined as dielectric with refractive index n=1.4 and the complex refractive index of ITO [36] was added to Lumerical material database as a table. Top-row is the side-view and bottom-row is the top-view *E*-field enhancement maps.

Qualitative insights regarding light absorption, scattering, and enhancement at the nanoscale level was numerically modeled using the finite difference time domain (FDTD) method (Lumerical, Ansys). The permittivity of ITO was calculated from experimentally measured complex refractive index $\tilde{n}$ [36] (Figure 7a). Cross-sections of light absorption $\sigma_{ab}$, scattering $\sigma_{sc}$, and extinction (total loss) $\sigma_{ex}\equiv \sigma_{ab}+\sigma_{sc}$ was calculated using a total-field scattered-field (TFSF) light source (Figure 7b) for typical nano-disk pairs of 250 nm and 230 nm diameters with 40 nm gap. The light field intensity in the nanogap and at the rim of ITO-disk at the top plane and the ITO-glass interface showed strong field enhancements E>10 (see insets in Figure 7b). Since ITO is transparent within the visible spectral range, a considerable light field enhancement can be created at the air-ITO and ITO-glass interface regions. This is also facilitated by the high refractive index of ITO n>1.5 at shorter wavelengths. The almost linear dispersion of ITO at visible wavelengths (Figure 7a) can be tapped for wavelength-specific light localisation on nanoparticles and metasurfaces. Figure A1 shows the light field enhancement for periodic patterns of nano-disk pairs on a square lattice with a Λ=1μm period under linearly polarised illumination with |E|=1. Apart from the expected enhancement inside the gap, also the case for plasmonic nanoparticles, a strong light field localisation on the top-surface as well as at the interface between ITO and glass was observed due to the transparency of ITO. This trait, along with its high electrical conductivity can be harnessed with a combination of optical and electrical modalities for characterisation of photo-electrochemical processes, as was shown for surface-enhanced Raman scattering (SERS) using interdigitated micro-electrodes with nanogaps [43]; see Appendix A and Figure A1 for E-field enhancement maps at different wavelengths.

## 3. Conclusions and Outlook

The use of a-ITO as a solid resist (negative type) was demonstrated using fs-laser pulses (230 fs/515 nm) tightly focused with an NA=0.9 objective lens onto a ⊘≈700 nm focal spot. Sub-diffraction limited disks with diameters ranging between 200–300 nm can be formed via multi-pulse exposure. The accumulation effect, which showed changes in disk diameter based on the number of pulses per burst, was revealed with a larger exponent S=1.5 at lower pulse energies $E_{p}\approx 0.5$ nJ. Such nano-disks can be used to define patterns of nanoparticles as well as to write micro-lines with widths considerably smaller than the diffraction limit. EDS confirmed the change of a-ITO into a poly-crystalline form, including a partial loss of In after laser exposure and development in 1% HF solution.

Combining the intricate control over re-crystallisation of a-ITO into c-ITO with glass surface re-melting and nano-dome formation [44], deposition and nano-writing of c-ITO over 3D complex surfaces such as black-Si [45], and atomic layer deposition (ALD) should open the door for applications concerning 3D nanoscale ITO metasurfaces and will be the focus of future research endeavours.

Low fluence exposure and highly localised oxidation of Si using ultra-short laser pulses was shown to act as a negative-tone resist for subsequent plasma etching [46]. The new possibility of using pre-surface modification on thin films and bulk materials as negative (or positive) resists for wet or dry etching is not well explored. This presents a promising area of maskless direct-write nanotechnology, a concept that is also explored in this study. The native scarcity of In helps motivate the search for other transparent conductive oxides, e.g., F-doped SnO_2_ (FTO) and AZO or Al-doped ZnO. Their patterning via direct laser-writing warrants further investigations.

## 4. Experimental: Samples and Procedures

The substrates used in the experiments were n-type amorphous a-ITO film deposited by radio-frequency (RF) sputtering and were ∼60 nm in thickness; purchased from Jinan Delta Optoelectronic Technology Co., Ltd., Shandong Province, China. Typical sheet resistance of the ITO film on glass deposited by magnetron sputtering was $R_{s}\equiv \rho /t=100\;\Omega /□$ for t=45±5 nm ITO thickness [47]. This is similar to commercially used samples [48]. For example, a coating of 125±2 nm ITO can be deposited by radio-frequency (RF) magnetron sputtering (KJL Axxis, Jefferson Hills, PA, USA) under the conditions: $<5\times 10^{-8}$ Torr chamber base pressure, 3 mTorr argon pressure during deposition, 120 W RF power over a 3-inch diameter target, substrate rotation 5 rpm, deposition duration 1050 s. Resulting film thickness was evaluated by scanning electron microscopy (SEM) (Raith 150two, Germany) via profiling of a cleaved-deposition control silicon chip.

Fabrication was carried out with a femtosecond (fs)-laser (Pharos, Light conversion) with $t_{p}=230$ fs pulse duration and λ=515 nm wavelength at a f=5 kHz repetition rate. The laser beam was focused using an objective lens with a numerical aperture NA=0.9 (Olympus). Pulse energy was measured by a power meter (Thorlabs) with accuracy of 0.01μW at 10 kHz repetition rate and the energy per pulse $E_{p}$ (on the sample) was calculated from the power/energy measured in front of the objective lens; transmission of the focusing optics was carried out in a separate experiment. Fabrication was controlled across high-precision mechanical stages (Piezo stage, Physik Instruments); experiments were conducted under ambient conditions. Linearly polarised fs-laser pulses were irradiated onto the surface of samples using an average laser fluence of (0.1–0.3) J/cm${}^{2}$, which was well below the threshold of glass-substrate ablation ∼2 J/cm${}^{2}$.

After laser exposure, modified ITO on glass substrates were soaked in a 1% vol. HF solution for 5 s then rinsed with water to remove the remaining HF solution. Surface morphology was observed via scanning electron microscopy (SEM; JSM-6700F, JEOL) and atomic force microscopy (AFM; Bruker). Energy-dispersive X-ray spectroscopy was carried out using an SEM microscope at 20 kV electron acceleration voltage. Finite Difference Time Domain (FDTD) simulations were carried out using Ansys Lumerical version 2022 R1.

## Data Availability

The data presented in this study are available on request from the corresponding author.

## Appendix A. Additional Simulations

Light field enhancement for a pair of ITO nano-disks in a periodic pattern (Figure A1). Examples of selected light field distributions showing the typical nano-gap enhancement mode as well as those related to the transparency of ITO. Fabry-Pérot and Fano type resonances can be explored with such transparent and electrically conductive ITO metasurfaces, including the design of perfect absorber metasurfaces [49]. The enhancement values are smaller within the UV-visible spectral range (Figure A1) where extinctions $\sigma_{ab,sc}$ are an order of magnitude smaller compared to the resonance at ∼850 nm (Figure 7). The Metal-Insulator-Metal (MIM) surfaces can be made using ITO as metal-base plate and nano-disk at the top layer with a dielectric of choice as I-layer in the MIM metasurface.
